# Supplementary material for: Identification of photoperiod-induced specific miRNAs in the adrenal glands of Sunite sheep (Ovis aries)
Source: Front Vet Sci. 2022 Jul 22;9:888207. doi: 10.3389/fvets.2022.888207 (PMC9354845; doi:10.3389/fvets.2022.888207)
Supplement: Supplementary file 1 [file Data_Sheet_1.ZIP › Supplementary file/miRDeep2 file.zip!/miRDeep2 file/Know_Structure/LP42A3/oar-mir-431.pdf]

miRBase precursor : oar-mir-431
Total read count : 448
oar-miR-431 read count : 49
remaining reads : 399

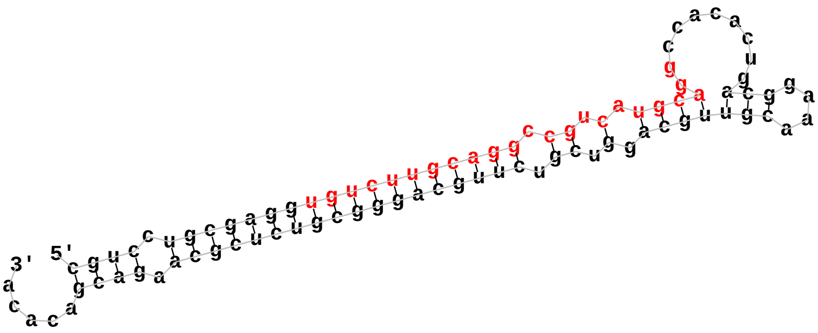

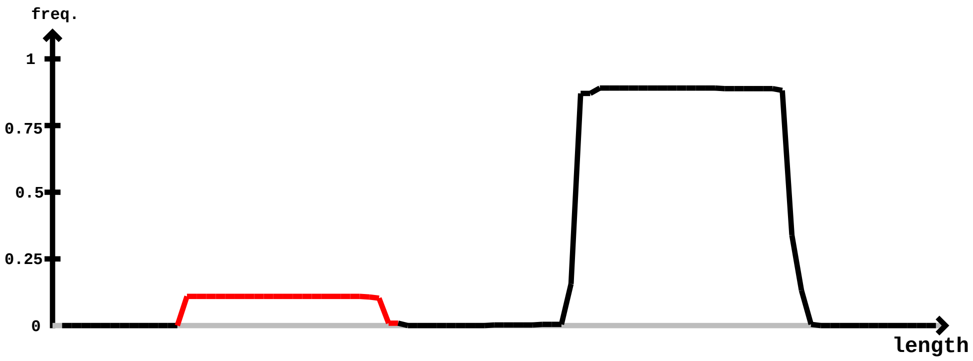


```
               oar-miR-431
5'- cguccugcgaggugucuugcaggccgucaugcaggccacacugacggaaacguugcaggucgucuugcagggcgucucgcaagacgacaca   -3'    exp
    ((((.((((((((((((((((((.((.(.((((..........(((....))))))).).)).)))))))))))))))))).)))).....     reads    mm    sample
    ............ugucuugcaggccgucaug............................................................     1        0     seq
    ............ugucuugcaggccgucaugc...........................................................     2        0     seq
    ............ugucuugcaggccgucaugca..........................................................     42       0     seq
    ............ugucuugcaggccgucaugcagg........................................................     4        0     seq
    ............................................cggaaacguugcaggucgucuugc.......................     1        0     seq
    .................................................acguugcaggucgucuugcagggcguc...............     1        0     seq
    ....................................................uugcaggucgucuugcagggcg.................     1        0     seq
    ....................................................uugcaggucgucuugcagggcgu................     48       0     seq
    ....................................................uugcaggucgucuugcagggcguc...............     10       0     seq
    ....................................................uugcaggucgucuugcagggcgucu..............     9        0     seq
    .....................................................ugcaggucgucuugcagggcg.................     2        0     seq
    .....................................................ugcaggucgucuugcagggcgu................     191      0     seq
    .....................................................ugcaggucgucuugcagggcguc...............     77       0     seq
    .....................................................ugcaggucgucuugcagggcgucu..............     48       0     seq
    .....................................................ugcaggucgucuugcagggcgucuc.............     2        0     seq
    .......................................................caggucgucuugcagggcgu................     4        0     seq
    .......................................................caggucgucuugcagggcguc...............     5        0     seq
```
